# If You’re a Rawlsian, How Come You’re So Close to Utilitarianism and Intuitionism? A Critique of Daniels’s Accountability for Reasonableness

**DOI:** 10.1007/s10728-017-0343-9

**Published:** 2017-03-22

**Authors:** Gabriele Badano

**Affiliations:** Centre for Research in the Arts, Social Sciences and Humanities, and Girton College, University of Cambridge, Cambridge, UK

**Keywords:** Healthcare resource allocation, Accountability for reasonableness, Public justification, Norman Daniels, John Rawls

## Abstract

Norman Daniels’s theory of ‘accountability for reasonableness’ is an influential conception of fairness in healthcare resource allocation. Although it is widely thought that this theory provides a consistent extension of John Rawls’s general conception of justice, this paper shows that accountability for reasonableness has important points of contact with both utilitarianism and intuitionism, the main targets of Rawls’s argument. My aim is to demonstrate that its overlap with utilitarianism and intuitionism leaves accountability for reasonableness open to damaging critiques. The important role that utilitarian-like cost-effectiveness calculations are allowed to play in resource allocation processes disregards the separateness of persons and is seriously unfair towards individuals whose interests are sacrificed for the sake of groups. Furthermore, the function played by intuitions in settling frequent value conflicts opens the door for sheer custom and vested interests to steer decision-making.

Norman Daniels is a key theorist in the field of justice and health. In particular, his account of fair process in healthcare resource allocation, which constitutes the main focus of my argument, is highly influential also beyond theoretical debates. It has been used as a guide to policy-making on multiple occasions by, for example, the British NHS, the Mexican government and the WHO.^1^

Daniels’s account of fair process, called ‘accountability for reasonableness’ (AFR), is the subject of much critical debate [1, 7, 9, 12, 24]. However, no commentator appears to take issue with Daniels’s [4, pp. 29–30] belief that his theory constitutes an extension of John Rawls’s hugely influential general theory of justice into the realm of health. In fact, much work in this area starts from the assumption that, like the rest of Daniels’s theory, AFR provides a faithful translation of Rawls’s account [7, 24].

This paper aims to demonstrate that AFR is vulnerable to important arguments advanced by Rawls. However, its interest is not limited to those who start from a commitment to Rawls’s theory of justice. Besides playing a fundamental role in Rawls’s account, the arguments that I intend to draw on are compelling in their own right and very relevant to healthcare resource allocation. My goal is to build upon these arguments to develop an original critique of AFR.

After reconstructing AFR, I draw on Rawls to argue that Daniels’s failure to keep a safe distance from both intuitionism and the aggregative logic of utilitarianism severely damages his theory of fairness in healthcare resource allocation. Next, I briefly outline a future research direction that could be explored in attempting to revise AFR, namely a shift towards a different form of public justification liberalism.

## Daniels’s Model of Fair Process

AFR is connected with Daniels’s analysis of the value of health. Daniels believes that health protects a person’s range of opportunities to pursue life plans. Rawls’s theory, along with several competing accounts of justice, provides reasons to protect opportunities and distribute them in an egalitarian fashion. Given that healthcare protects health, Daniels [4, pp. 29–78] maintains that healthcare should be regarded as special, which means that societies should provide universal access to it, in isolation from ability to pay and other social goods.

As important as the specialness of healthcare may be when it comes to organising healthcare systems at a general level, Daniels recognises that no principle of opportunity, Rawlsian or otherwise, is fine-grained enough to provide answers to the specific substantive questions that make up the routine of healthcare resource allocation agencies. Numerous substantive criteria are generally considered to be suitable for governing the allocation of scarce healthcare resources, while available theories of opportunity are too abstract to determine how these criteria should be traded off against each other when they conflict. Daniels lists three particularly important conflicts as representative of all others. How much priority for the sickest is justified vis-à-vis the production of greater aggregate health benefits? When should significant health benefits to a smaller number of persons be outweighed by the aggregation of more modest benefits to a larger number of persons? How should the value of a fair chance to derive some benefit from available resources be balanced against more cost-effective interventions? From the perspective of available theories of opportunity, a wide range of possible answers to each of these questions appear equally just [4, pp. 103–110].

To solve these conflicts, the principle of opportunity needs to be supplemented. Drawing on Rawls’s notion of pure procedural justice, Daniels claims that resource allocation decisions should be regarded as just when they result from a fair decision-making process, where fairness must be understood in terms of the four conditions constituting AFR:

- Publicity: Decisions and supporting rationales must be transparently stated.
- Relevance: ‘The rationales for limit-setting decisions should aim to provide a *reasonable* explanation of how the organization seeks to provide “value for money” in meeting the varied health needs of a defined population’. An explanation is reasonable if it is grounded in considerations that can be accepted *as relevant* by persons who are willing to provide justifications for the allocation of resources they support.
- Revision and appeals: Mechanisms must be in place to challenge decisions.
- Regulation: There must be uniform enforcement of the other three conditions.^2^

Relevance, which is supposed to constrain the substance of the reasoning leading to decisions, is the primary target of this paper’s criticism. Relevance is very inclusive towards the substantive criteria that may be proposed as suitable for governing resource allocation. Indeed, a wide variety of criteria can be considered to have at least *some* relevance to the pursuit of some unspecified ‘value for money’ in meeting health needs. This leads to decision-makers adopting long lists of relevant criteria, as reflected in the practice of those real-world resource allocation agencies that apply AFR.

Consider the British National Institute for Health and Care Excellence (NICE), which not only endorses AFR, but is also typically described by Daniels [5, pp. 178–180] as a successful application of AFR’s key ideas. Founded in 1999 and operating at arm’s length from the Department of Health, NICE provides guidance in a number of areas, but is most often discussed for its compulsory recommendations on the coverage of pharmaceuticals and other health technologies in the NHS. Over time, NICE has progressively introduced a number of so-called ‘equity weightings’ to be balanced against the cost-effectiveness of health technologies to decide whether they should be funded.

To be sure, cost-effectiveness analysis (CEA) still plays a uniquely important role in NICE’s process, in that equity weightings are only considered when the cost-effectiveness of a technology falls below a certain mark and, therefore, NICE needs reasons other than cost-effectiveness to justify a positive recommendation; beneath an even lower mark, the support provided by the equity weightings must be exceptionally strong for that technology to be funded despite its poor cost-effectiveness. Still, when the conditions are right, decision-makers can appeal to severity of disease, the potential for innovation of the technology under appraisal, stakeholder persuasion, the premium placed on benefits accruing to patients at the end of their lives, the extra priority for the members of disadvantaged groups and the special attention to be paid to children [17, 20]. In a recent consultation paper, NICE [18] proposes that the wider societal benefits of technologies should be added to the list, and it is hard to see why this proposed criterion (and many others that could have been suggested with it) should be excluded if the question is merely one of relevance to the pursuit of value for money in meeting health needs.

To prepare the ground for my critique of Daniels, it is important to discuss CEA in greater detail. CEA is an aggregative criterion in that it combines the health gains and losses of different individuals into the health gain and loss of a group as a whole; its basic idea is that decision-makers should allocate available funds so as to create the greatest sum total of health benefits *aggregated across the population*. Health benefits are generally measured in terms of quality-adjusted life years (QALYs), which integrate life expectancy and health-related quality of life. To see how efficiently a certain intervention can foster the maximisation of aggregate benefits in the context of a limited budget, the cost of the intervention is divided by the number of QALYs that would be created by it. This gives the cost of the intervention per QALY added to the health of the population; the lower the cost per QALY, the greater the cost-effectiveness of an intervention [2, pp. 53–78].

Cost-per-QALY estimates for interventions are widely used, generally in conjunction with other criteria, to determine which interventions should and should not be funded. Daniels [4, p. 114] makes it clear that the three conflict cases, noted above, that he uses to justify AFR demonstrate that ‘CEA by itself cannot serve as a decision procedure’ for allocating healthcare resources. However, the exposition of his theory of AFR attaches great importance to cost-effectiveness—perhaps greater importance than that attached to any other relevant criterion. To see how, let us go back to the three conflict cases.

Although priority to the sickest, the premium placed on individual ability to benefit from intervention and the provision of fair chances may well clash with each other, none of Daniels’s conflict cases pits two of these quintessentially distributive considerations against one another. Each of Daniels’s cases, which are paradigmatic examples of the conflicts that AFR is meant to arbitrate, opposes the aggregative and maximising logic of CEA against a different consideration that stresses the importance of who receives the benefits. This suggests that an implicit assumption underlying AFR is that resource allocation processes have two high-order goals, which must be balanced: the maximisation of aggregate population health and the distribution of benefits fairly.^3^ Given that cost-effectiveness is one and the same as the former goal, virtually all the other relevant considerations are grouped together under the latter goal, highlighting an asymmetry between CEA and any other relevant criterion in the theory behind AFR.

As further support to the claim that CEA is not simply a relevant consideration among others, it is important to recall that Daniels defines the relevance condition as relevance to the goal of creating value for money. Given CEA’s commitment to creating as much good as possible from the money available for healthcare, the notion of value for money is commonly associated with CEA, to the point that this notion is sometimes almost reduced to cost-effectiveness [17, p. 4]. Again, it appears that the theory behind AFR has a particularly close link with the idea of cost-effectiveness.

## Two Problems with Aggregation

My reconstruction depicts AFR as a conception of fair process in which decision-makers must allocate resources on the basis of cost-effectiveness calculations balanced against a wide variety of relevant countervailing considerations. In the introduction, we saw that Daniels and his commentators seem to agree that AFR works well as a supplement to Rawls’s general theory of justice. My critique of AFR is prompted by the sense that they are missing something important.

Rawls [22, pp. xvii–xviii] clearly states that the main aim of his theory is to put forward a superior alternative to the only approaches to the allocation of societal resources that philosophers deemed viable in the 1960s, namely utilitarianism, intuitionism and, most appealing of all, a mix of them in which the principle of utility is restricted by intuitionistic constraints. This aim is grounded in compelling arguments against utilitarianism and intuitionism. My goal in this section and the next is to demonstrate that these arguments can be used to show that AFR is a flawed account of fairness in healthcare resource allocation. Indeed, when Rawls’s arguments are adapted to the case of AFR, it will emerge that Daniels’s model looks much like the mixed approach that Rawls wishes to find an alternative to.

Consider first Rawls’s [22, pp. 19–30] argument against utilitarianism, which is the general view that societal resources should be allocated so as to maximise the sum total of satisfaction aggregated throughout all members of society. Rawls’s argument can be thought of as consisting of two closely connected parts. To start with, Rawls argues that utilitarian institutions violate the separateness of persons. A single individual is free to impose a loss on herself in order to secure a greater gain, perhaps at a later date. However, utilitarianism requires that the losses imposed on *certain* individuals should be freely balanced against the gains accrued to *others*, therefore treating society as though it was a single person, produced through the conglomeration of all its members.

Given that CEA requires that the health losses to some be balanced against the health gains to others so as to maximise aggregated health benefits, CEA is affected by the same problem. Insofar as decision-makers employ CEA, the health gain and health loss of a social conglomerate influence resource allocation decisions in their own right, effectively making such a conglomerate into a somewhat monstrous independent unit of concern, above and beyond the concern due to individual members of society.

Also the second part of Rawls’s argument targets an element that utilitarianism shares with CEA, namely the exclusive concern for the maximisation of aggregated benefits, as opposed to their distribution. If either utilitarianism or CEA plays any role in allocating limited resources, there will be cases in which decision-makers assign priority to giving a smaller benefit to each member of a larger group over a larger benefit to each member of a smaller group. The larger the role either utilitarianism or CEA is allowed to play, the greater the sacrifices that individuals from the smaller group will be required to make in these sorts of conflict cases. According to Rawls, it is highly problematic to require that individuals make important sacrifices specifically *for the sake of a group*, as opposed to making important sacrifices because one or more other individuals have a stronger claim to available resources. The problem is the violation of the compelling idea, derived from the social contract tradition, that a just society is ultimately built on equal respect and concern *for individuals*, who enjoy a form of inviolability by the claims of groups as such.

A supporter of CEA could try to deflect my criticism by objecting that utilitarianism and similarly aggregative views are actually built on a separate concern for each person. As claimed by Hirose [10], this commitment to the separateness of persons is reflected in the principle that the well-being of everyone should count for one and no more than one for the purposes of the utilitarian calculus.^4^ It is unclear to me how the principle that the well-being of everyone should count for one in an interpersonally aggregative calculus expresses a commitment not only to impartiality between competing interests, but also to the separateness of persons, especially in the relevant moral sense of treating them as separate ultimate units of concern. Hirose [10, p. 196] anticipates this reaction, and he briefly comments that impartiality logically implies separateness; utilitarianism cannot be impartial between the well-being of Annie and Betty ‘unless it acknowledges the fact that Annie and Betty live different lives’.

However, this alleged logical relation linking impartiality *between interests* with the separateness *of persons* does not withstand scrutiny. A person can accept for herself a principle of rational choice requiring that the satisfaction of each of her interests should count for one (regardless, for example, of whether they qualify as higher or lower pleasures in a Millian sense) without transforming them into interests that, instead of all being part of her life plan, belong to different persons—and, moving close to the moral understanding of separateness, without taking the satisfaction of any of her interests to enjoy an inviolability that cannot be outweighed by any aggregation of other individually weaker interests of hers.

How damaging to Daniels is this Rawlsian-inspired twofold critique of CEA? The section ‘Daniels’s Model of Fair Process’ explained that when presenting his theory of AFR, Daniels frames his arguments in a way that effectively gives a place of honour to the idea of cost-effectiveness. This already demonstrates Daniels’s failure to fully appreciate the strength of Rawls’s arguments against utilitarianism and their relevance to CEA. However, this is by no means all that can be said against Daniels. AFR also imposes too few constraints on the extent to which CEA can govern *the practice* of resource allocation, therefore condoning seriously unfair decision-making processes.

To be sure, I noted earlier that Daniels rejects the view that CEA should serve by itself as a decision procedure. However, AFR does *not* exclude processes for allocating resources that assign a high, albeit not absolute, priority to cost-effectiveness in its conflicts with distributive considerations. To give a concrete example of such processes, we saw that Daniels typically depicts NICE as a successful application of AFR’s key ideas, despite the especially important role that, as mentioned in the section ‘Daniels’s Model of Fair Process’, CEA plays in NICE’s procedures.

Consequently, AFR condones processes that are seriously flawed (according to the Rawlsian line of thought that I have developed in this section) by virtue of the large use of CEA they make and, therefore, by virtue of the great extent to which they are affected by the two problems with the aggregative logic of CEA. Indeed, if a resource allocation process decides in favour of cost-effectiveness in a wide range of conflict cases with the various countervailing considerations, (1) a great deal of the reasoning at the core of such a process is defective because it is built upon a misguided unit of concern, and (2) the process is seriously unfair towards those potential beneficiaries who are now required to sacrifice considerable individual claims simply for the sake of a group.

As a last defence of AFR, one might distinguish AFR itself (strictly understood as the framework made up of the core notions of publicity, relevance, revision and appeals, and enforcement) from the way in which Daniels presents and develops it. Next, it might be suggested that in itself, AFR is not necessarily vulnerable to my Rawlsian-inspired arguments against cost-effectiveness, in that CEA could simply be excluded *as irrelevant* to healthcare resource allocation based precisely on Rawls’s objections to aggregation. My response to this ingenious way of moving beyond Daniels is that it stretches the concept of relevance too thin. The problems with aggregation identified by Rawls are not problems of irrelevance to the pursuit of value for money in the allocation of scarce resources. Therefore, the notion of relevance is simply ill-suited to narrowly constrain the use of cost-effectiveness. In turn, this means that AFR should be replaced by an account of fair process that has the necessary resources to impose stricter constraints on CEA, so as to exclude the serious instances of unfairness overlooked by AFR. To identify another weakness in this model, let us now discuss Rawls’s argument against intuitionism.

## The Case Against Intuitionism

According to Rawls’s definition, intuitionists believe that (a) a plurality of irreducible substantive values apply to political issues and (b) there is no explicit principle for weighing such values against each other. Why is this approach called ‘intuitionism’? If a plurality of values apply to political issues, they will often conflict with one another. Given that there is no explicit principle for balancing values in all conflict cases or, at least, confining intractable value conflicts within narrow limits, intuitions are bound to greatly influence decision-making by determining how conflicts must be settled.

Rawls points out that intuitionism is particularly tempting when the focus is on specific public policy areas such as fair wages and—we may add—healthcare resource allocation. I argue that AFR yields to this temptation, effectively proposing an account of fair process in which cost-effectiveness is intuitively balanced against a plurality of other substantive criteria. The section ‘Daniels’s Model of Fair Process’ established the link between Daniels’s relevance condition and long lists of criteria. Moreover, Daniels’s case for AFR demonstrates that, according to him, explicit principles for weighing those criteria against each other are unavailable; we need AFR precisely because available theories of opportunity cannot explain how to balance CEA against the numerous other criteria that appear to be suitable for governing resource allocation. Consequently, decision-makers following AFR are bound to make frequent use of intuitions when cost-effectiveness conflicts with other relevant criteria.

What is the problem with the work done by intuitions in settling value conflicts? Intuitions are opaque in the sense that a person cannot be expected to satisfactorily explain to others why her intuitions favour one possible solution to a value conflict over others. Hence, Rawls [22, pp. 30–36] maintains that *vested interests and sheer custom* are free to hide behind intuitive judgements to determine the solutions to value conflicts in a way that is virtually impossible to detect. The risk is that sheer power and status-quo bias hijack decision-making without even being detected.

Rawls’s argument against intuitionism is particularly relevant to healthcare resource allocation decisions because of the context in which such decisions are made. This context, which I will now briefly discuss, makes it all the more likely that the use of an intuitionistic approach such as AFR ends up serving as a smokescreen for status-quo bias and, more importantly, for vested interests to steer the decision-making. This result violates the very notion of fairness that Daniels wishes to place at the basis of AFR, namely fair process as a transparent exchange of reasons in the search for resource allocation arrangements that truly guarantee value for money spent.

Agencies responsible for allocating healthcare resources are on the receiving end of a huge amount of pressure exerted by multiple lobbies. To cite but a few examples, the enormous lobbying power of pharmaceutical industries is always at work to loosen the constraints on drug coverage that resource allocation agencies impose in the attempt to stay within their budgets. The interests of Big Pharma generally converge with the interests of patient advocacy groups, while the media constitute another important actor, which has traditionally been keen to launch campaigns against resource allocation efforts. On top of all this, elected politicians often have incentives to side with such lobbies. In sum, as claimed by Williams et al. [30, p. 90], ‘the interplay of interest group agendas is nowhere more significant than in healthcare’.^5^

As an example of the pressure exerted by lobbies, consider the case of Herceptin in the UK. As explained by Ferner and McDowell [6, p. 1269], Herceptin well exemplifies the ability of pharmaceutical companies to make the general public attuned to a promotional message about a drug long before licencing, through enthusiastic press releases and exhortations to spread the word, delivered as soon as positive results start to emerge from early trials. In 2005, the drug had been used for a few years to treat advanced breast cancer under the NHS, and pressure mounted on the NHS after positive results in the treatment of early-stage breast cancer had started surfacing. Newspapers published numerous stories, attacking what was depicted as red tape that was denying many women access to a wonder treatment. Patient advocacy groups did their part, with one of them marching on Downing Street in September 2005 to submit a petition.

Local commissioning authorities, at the time called ‘primary care trusts’ (PCTs), were ultimately responsible for choosing whether NHS providers in their area should start offering Herceptin to early-stage breast cancer sufferers. At that stage, the European Medical Agency had not yet received the necessary information to assess the safety of Herceptin in the treatment of early-stage breast cancer in order to issue a licence. Thus, PCTs were pressurised into making coverage decisions not only before NICE could appraise value for money, but also before safety issues could be assessed. Nonetheless, politicians went to great lengths to ensure that as many PCTs as possible would cover Herceptin. In a Department of Health press release, the Secretary of State for Health, Patricia Hewitt, declared that she wanted to see Herceptin in widespread use. She went as far as to meet with the staff of one of the PCTs that had upheld the principle that the licensing process should not be bypassed—unsurprisingly, the decision taken by the PCT was reversed after the meeting [6, 28, pp. 1–9].

We can now appreciate the full potential for damage that the intuitionistic approach embedded in AFR is likely to inflict upon the fairness of healthcare resource allocation processes. The Herceptin case is only a particularly egregious example of the sort of pressure that, as encapsulated in the words of Williams and colleagues, vested interests routinely put on resource allocation. If we accept that a plurality of values apply to resource allocation and only intuitions can settle their conflicts, decision-makers are offered the ‘easy’ option of giving in to that pressure while also obfuscating the fact that vested interests are effectively governing the decision-making.

Daniels himself stresses that a great deal of disagreement exists, among both theorists and ordinary persons, about how to balance conflicting criteria for making decisions and answer specific healthcare resource allocation questions; many different orderings of criteria and many different decisions seem right to different persons. Therefore, if we exclude strikingly implausible arrangements, decision-makers following AFR often have the option of appealing to intuitions to justify an ordering of conflicting criteria that leads to a decision that favours the most powerful lobbies with an interest in the issue at hand. In sum, given the context in which healthcare resource allocation takes place, the intuitionistic nature of AFR creates a very high risk that powerful vested interests will steer decision-making without even being detected, violating Daniels’s own idea of fairness as transparent reason-giving by decision-makers in search of truly valuable resource allocation arrangements.

It is important to pause a little longer over the intuitionistic character of AFR, to forestall any misunderstanding of my argument. Readers might wonder whether my argument only works because it has narrowly focused on relevance, apparently forgetting about publicity and the other conditions of AFR. I have not forgotten about them, and I believe that transparent reason-giving can help considerably in the fight against status-quo bias and vested interests, as can be illustrated by going back to the Herceptin case. It is hard to imagine any local commissioner openly declaring that they have decided to fund Herceptin because they wish to please the pharmaceutical industry, or even because they simply want the Secretary of State and pressure groups off their back. Among other things, these sorts of rationales would have likely faced challenge had PCTs had any internal appeals process. Therefore, AFR is better suited to curb the influence of status-quo bias and vested interests than so-called systems of ‘implicit rationing’, where the processes through which healthcare resources are allocated are not publicly acknowledged.

However, precisely because I appreciate the importance of publicity in the justification of decisions, I believe that the intuitionistic character of AFR still creates a problem. The frequent *intractable* value conflicts that, as we have seen, AFR is meant to deal with create a space that is *by its nature closed to transparent reason*-*giving* and, in turn, to the protection transparency offers against sheer custom and vested interests. This feature of value conflicts that are taken to be intractable to explicit principles has been stressed both by critics and proponents of publicity. One of Mechanic’s [13, 14] argument for implicitly ‘muddling through’ healthcare resource allocation decisions is that, to strike the right balance among the many considerations relevant to the problem at hand, decision-makers often have to make judgement calls that, by their very nature, cannot be transparently explained to others. At the other end of the spectrum, Richardson [23, pp. 287 and 305, respectively] criticises intuitive balancing precisely because the grounds for accepting a certain ordering of conflicting values as intuitive will always be ‘mysterious’ from the perspective of others, and will never be ‘open to rational public debate’. It is through this opaque process for arbitrating value conflicts that status-quo bias and vested interests risk creeping back, at least in some measure, into decision-making procedures governed by AFR.

My discussion of Herceptin was meant to give a sense of the sheer amount of pressure faced by healthcare resource allocation decision-makers—a pressure so strong that it sometimes threatens the standing of resource allocation agencies in society, if not their prospects for survival [25, p. 23]. It is against this background, I reiterate, that we should assess the risks involved in AFR admitting long lists of values into decision-making while acknowledging that many different orderings of values and, therefore, many different resource allocation decisions seem right to different persons. The need to intuitively balance conflicting values will often create a chance for decision-makers to yield to that huge pressure by publicising as intuitive to them the ordering of relevant values that leads to the decision favoured by the most vocal or otherwise most powerful interest groups.

This problem constitutes a serious flaw in Daniels’s model. It is a problem that might not be completely solvable; as acknowledged by Rawls, it is implausible to completely eliminate intuitions from the process of adjudicating value conflicts. However, it is important to find a way to make the problem associated with intuitions less serious than it is under AFR by confining the use of intuitions within narrower limits. As sketched in the next section, an option worth exploring is to develop the notion of public justification beyond AFR’s conditions, in a way that imposes a tighter frame of reasoning on decision-makers.

## What Next?

This paper has shown that AFR is vulnerable to powerful arguments originally advanced by Rawls, leaving us with the task of developing a revised account of fairness in resource allocation that does more to limit the role of CEA and confines intuitions within narrower limits. This is an extremely complicated task, and I am forced to leave its completion for another day. However, I wish to briefly sketch a possible research direction that will be worth considering, perhaps among others, when examining how to revise AFR.

AFR’s problems are due to the relevance condition, whose inclusivity leads to long lists of criteria being admitted into decision-making and is hospitable towards procedures that make extensive use of CEA. The other conditions help ease those problems, at least regarding sheer custom and vested interests, but do not go far enough. Therefore, although publicity, revision and appeals, and enforcement should be retained, a fitting substitute should be found for relevance. Daniels himself [3, pp. 201–202] points us in an interesting direction when he clarifies that AFR incorporates a principle of universal acceptability among reasonable persons, but only in the ‘attenuated’ sense that everyone must be able to see the relevance of the rationales. He admits that there are ‘fuller’ conceptions of universal acceptability, which seem a promising place to look for candidates for replacing relevance.

A possible replacement, which embraces acceptability *without strings attached*, requires that decision-makers strive to ground resource allocation decisions in rationales that each reasonable person can accept, where reasonable persons are understood to be those who are themselves committed to decisions that everyone similarly motivated can accept. This requirement could be called the ‘full acceptability condition’, and closely resembles classic formulations of the duty of public justification for binding decisions,^6^ already brought to bear on issues of distributive justice by Nagel [16]. Also, this requirement is virtually identical to classic formulations of contractualism in the debate over the distribution of scarce benefits, as exemplified, once again, by Nagel and also by Scanlon’s [27] idea that decisions should be made according to principles that no one could reject in a situation in which everyone is committed to proposing principles that no other similarly motivated person could reject.

Thus far, I have only laid out the definition of the full acceptability condition. But how do its requirements differ from those imposed by relevance on resource allocation? Why is the full acceptability condition an option worth considering? First, it would impose limits on the use of CEA well beyond those set by AFR. Contractualists explain that when applied to the distribution of scarce benefits, the requirement to look for arrangements that everyone can accept (or no one can reject) imposes *a rather specific and considerably tight frame of mind on decision*-*makers*—one that asks them to carry out pairwise comparisons between the perspective of each potential beneficiary and that of every other, which in turn pull strongly towards a commitment to assigning priority according to the strength of the claims to resources that potential recipients of intervention can make as individuals. To see how this tight frame of reasoning is derived, recall that resource allocation decisions are bound to create winners and losers. Nagel [15, p. 123] points out that in these circumstances, no decision can be completely acceptable to everyone. Therefore, decision-makers committed to universal acceptability have to settle for the arrangement that is most acceptable to the person to whom it is least acceptable. Nagel suggests that the decision that is most acceptable to those to whom it is least acceptable should be identified through pairwise comparisons, with the aim of identifying which member of each pair has stronger grounds for rejecting a resource allocation arrangement that does not help her.^7^

What matters for the purposes of my argument is that, as contractualists make clear, *no interpersonal aggregation* is part of this reasoning method. The basic idea here is that aggregative and maximising principles can only satisfy acceptability *to a single point of view that combines all individual perspectives* into one, while this frame of reasoning aims for acceptability *to each individual perspective*.^8^

By themselves, AFR’s original conditions could not have imposed this tight non-aggregative frame of reasoning. The section ‘Two Problems with Aggregation’ already explained that the notion of relevance is ill-suited to place strict constraints on the use of aggregative principles. A similar point can also be made about publicity as understood by AFR, i.e., as disclosure of decisions and supporting rationales to the general public. It seems implausible to assume that the members of the public who are concerned with healthcare resource allocation are generally committed to the specific way of reasoning about it that involves placing oneself (at least schematically) in the shoes of each potential beneficiary, in order to identify who has the strongest claim to available resources. This commitment presupposes a strongly altruistic attitude, which is a lot to expect, especially in an area of debate where the members of certain patient groups have much to lose. Moreover, it presupposes a very specific way of giving shape to that attitude—one concerned with acceptability to each. Without any widespread and strongly-felt commitment of this sort in the real world, it seems a stretch to suggest that by itself, transparency could push decision-makers progressively closer to the anti-aggregative frame of reasoning that is integral to the universal acceptability condition.

Now, although free from aggregation, the reasoning method that is imposed by universal acceptability is usually proposed by contractualists as part of sophisticated theories, which include arguments suggesting that such a method converges on the same conclusions as CEA in certain cases where aggregative methods give intuitively right answers. Most notably, the non-aggregative reasoning imposed by the full acceptability condition is said to prioritise helping the greater number in conflict cases between differently-sized groups of otherwise similar potential beneficiaries [27, pp. 231–235; and 11, pp. 48–77]. Also, given that it seems fair to say that the strength of an individual’s claim depends in part on the extent to which she could benefit from intervention [15, p. 125; and 26, p. 123], non-aggregative reasoning appears to have an answer to the so-called ‘bottomless-pit problem’, posed by patients who are extremely badly-off, but only capable of receiving trivial benefits.

Moreover, the theories behind non-aggregative reasoning are sometimes so sophisticated as to argue that there are specific circumstances in which non-aggregative reasoning itself requires passing matters on to CEA or other aggregative methods, as in conflict cases between a smaller group of potential beneficiaries and a larger group with claims that, although weaker, are strong enough to remain relevant. Building on previous work by Kamm, Voorhoeve [29] argues that in these cases, non-aggregative reasoning cannot identify any arrangement that every reasonable person can accept, therefore abdicating the matter to aggregative reasoning. If considered together with the instances of convergence, would this limited role for CEA allowed by full acceptability be enough to create a plausible account of resource allocation? If not, would minor adjustments be sufficient? Also, are the arguments highlighting convergence with and a role for cost-effectiveness solid? These are some of the questions that a full evaluation of the full acceptability condition would have to tackle. On the face of it, however, this condition seems promising precisely because the problematic logic of aggregation would be much more rigidly constrained than under AFR.

The second reason why the full acceptability condition deserves attention concerns intuitions. We have just seen how precisely the reasoning method required by full acceptability dictates when aggregation is and is not allowed, going well beyond AFR’s laxer relevance and publicity conditions. This reduces to a minimum the need to resort to the intuitions of decision-makers to solve the conflict cases opposing cost-effectiveness (or any other aggregative criterion, for that matter) to any countervailing consideration, as in Daniels’s three paradigmatic conflicts. In all such cases, the full acceptability condition itself offers specific answers.

Of course, many criteria that are used to allocate healthcare resources do not involve aggregation, and they may conflict with one another. However, earlier in this section we saw that, in virtue of the tight frame of reasoning that full acceptability, but not relevance or transparency, imposes on decision-makers, full acceptability leads to a commitment to assigning priority according to the strength of individual claims. Consequently, a criterion should only be included in public justification if it can be represented as providing the basis for the claims of affected individuals to available resources. A hypothesis that seems worthy of future analysis is that the full acceptability condition would also exclude several criteria that, although not obviously aggregative, are nonetheless resistant to being represented as bases for individual claims. Simply by browsing NICE’s list of relevant criteria as described in the section ‘Daniels’s Model of Fair Process’, we come across the principle that extra priority should be assigned to technologically innovative drugs and the idea that drugs that stakeholders consider to be priorities should be given extra importance, independent of the support offered to them by other criteria. Criteria like these seem to satisfy Daniels’s relevance while being *impersonal* in the relevant sense, justifying further analysis that would seek confirmation that they cannot be recast as bases for individual claims and, therefore, that they should indeed be excluded from deliberation.

Given that fewer criteria create fewer opportunities for conflict, and fewer conflicts lead to a decreased need for intuitive balancing, a shortened list of criteria confines the use of intuitions within narrower limits. Although intuitions are far from eliminated, detailed instructions regarding aggregation and a shorter list of criteria than under AFR appear to reduce the volume of intuitive judgements involved and, therefore, the risks associated with their being by nature closed to public scrutiny.

## Conclusion

In the previous section, I suggested that full acceptability seems worthy of attention. From the perspective of this paper, however, the merits of full acceptability or any other specific alternative to relevance are secondary; my main goal has been to argue against AFR, demonstrating that we must search for a revised account of fairness that somehow imposes stricter constraints on CEA and confines intuitions within narrower limits.

Going back to the question asked by the title of this paper, it is not difficult to understand why Daniels proposes a theory that has so much in common with the two main critical targets of Rawls’s theory of justice. Certainly, it has not been my intention to suggest that Daniels has not paid enough attention to Rawls’s arguments. Rather, Daniels appears to be interested in providing a framework for the allocation of resources by often unRawlsian actual persons, many of whom place considerable weight on cost-effectiveness and take long lists of values to be relevant to resource allocation. This interest is, of course, fully understandable. However, by reconstructing Rawls’s arguments, and by bringing them closely to bear on healthcare resource allocation, I have aimed to flesh out the full extent of the damage suffered by AFR in the process of accommodating real-world tendencies. Therefore, my conclusion is that Daniels has been too generous to such tendencies, and that theorists should now put greater effort into understanding the direction in which they should be reformed.

## Footnotes

1 For the NHS, see NICE [17]. For Mexico and the WHO, see Daniels [4, pp. 274–296].

2 Daniels [4, pp. 117–133, while the direct quotation of the relevance condition is from page 118, with emphasis in the original]. AFR draws on the work that Daniels has carried out with Sabin [5].

3 An explicit reference to the conflict between maximisation and distribution is sometimes used by Daniels to frame the problems facing the application of AFR to real-world resource allocation. For example, see Daniels [4, pp. 253–254 and 303–304].

4 See also Norcross [19, pp. 79–80].

5 See also Goddard et al. [8].

6 This duty is most famously captured by the theory of public reason proposed by Rawls [21, pp. 212–254].

7 See also Nagel [16, pp. 63–74] and Scanlon [26, pp. 119–123].

8 Nagel [15, p. 86]. In the same passage, Nagel also rightly notes that a ‘schematic’ rendering of individual claims, which can therefore be considered ‘in essentials’, would suffice. In the interest of practicality (and without involving any interpersonal aggregation), it would therefore be admissible to create, for example, a prioritised list that ranks healthcare interventions based on the strength of the claims that *typical* individual members of different patient groups can make to them, ignoring certain differences among individual members of the same group or sub-group of patients.
